# Exploring the Role of Phytochemical Classes in the Biological Activities of Fenugreek (*Trigonella feonum graecum*): A Comprehensive Analysis Based on Statistical Evaluation

**DOI:** 10.3390/foods14060933

**Published:** 2025-03-09

**Authors:** Rizwan Ahmad, Aljawharah Alqathama, Riyad Al-Maimani, Hamdi M. Al-Said, Sami S. Ashgar, Mohammad Althubiti, Naif A. Jalal, Majed Khan, Mutaz Algarzai

**Affiliations:** 1 Department of Natural Products, College of Clinical Pharmacy, Imam Abdulrahman Bin Faisal University, Dammam 31441, Saudi Arabia; majedskhan@gmail.com (M.K.); m.garzai@outlook.com (M.A.); 2 Department of Pharmaceutical Sciences, College of Pharmacy, Umm Al-Qura University, Makkah 21955, Saudi Arabia; aaqathama@uqu.edu.sa; 3 Department of Biochemistry, Faculty of Medicine, Umm Al-Qura University, Makkah 21955, Saudi Arabia; ramaimani@uqu.edu.sa (R.A.-M.); mathubiti@uqu.edu.sa (M.A.); 4 Department of Microbiology and Parasitology, Faculty of Medicine, Umm Al-Qura University, Makkah 21955, Saudi Arabia; hmibrahim@uqu.edu.sa (H.M.A.-S.); ssashgar@uqu.edu.sa (S.S.A.); najalal@uqu.edu.sa (N.A.J.)

**Keywords:** fenugreek, cytotoxicity, antimicrobial, α-amylase, statistical models, correlation

## Abstract

Background: This study encapsulates an in-depth correlation analysis for the biological activities (cytotoxicity, antimicrobial, and α-amylase inhibition) vs. the phytochemical classes (flavonoids “FV” and alkaloid “AL”) present in fenugreek seed extract. Methodology: Cell cultures for different cell lines were used to assess the cytotoxicity and selectivity (IC_50_ value), agar diffusion assay was used to determine the MIC and MBC for different bacteria and fungi, whereas α-amylase inhibition was studied to evaluate the antidiabetic potential for the forty-five different origins of fenugreek seed extracts. An in-house analysis for the phytochemical classes of flavonoids (rutin, RT; quercetin, QT; luteolin, LT; kaempferol, KF) and alkaloid (trigonelline, TG) was performed for the seed extracts. Results: A lower IC_50_ value (14.7 ± 1.46 µg/mL) was recorded for the IR3M extract against the HT29, MCF7 (13.03 ± 1.95 µg/mL), and MRC5 (14.58 ± 2.92 µg/mL) cell line. The extract with the lower IC_50_ value (8.17 ± 0.73 µg/mL) against HepG2 was IR2M. For the antimicrobial activity, a lower MIC value (6.3 mg/mL) was observed for E2C, E2M, E3C, and I3H extracts against SF and for the E1M, Y3C, IR2H, IR3H, and IR3C extracts against SA. The lowest MBC value (12.5 mg/mL) was seen for E2C, E2M, E3C, and I3H against SF as well as for the extracts E1M, Y3C, IR2H, IR3H, and IR3C against SA. The extracts of Q1H (49.07 ± 2.45 µg/mL) and Y3C (43.65 ± 2.97 µg/mL) exhibited IC_50_ values comparable to the standard drugs tested for α-amylase inhibition. The statistical models were of Pearson’s correlation. Principal component analysis (PCA) and a paired *t*-test established a strong positive correlation for the FV (QT, KF, LT) and alkaloid (TG) (*p* < 0.05) in the biological activities (cytotoxicity, antimicrobial, and α-amylase inhibition), thereby suggesting a substantial role for these phytochemical classes in the traditional and medicinal uses of fenugreek seeds. Conclusions: The FV and alkaloid are the key to impart the biological properties to the fenugreek seeds, hence their presence is utmost in the fenugreek seeds. This research work may be used as marker to help authenticate the fenugreek seeds for the quality variation in the major phytochemical classes.

## 1. Introduction

Fenugreek (*Trigonella foenum graecum*) is an annual plant with significant nutritional and pharmacological value. The leaves and seeds of the fenugreek plant are used in cooking where it confers a distinct smell along with the color and texture of food [1]. Fenugreek has been used for centuries for its laxative, demulcent, galactagogue, expectorant, and carminative properties, and the herb/spice features in pharmacopeia worldwide, including in the Ayurvedic, Chinese, Arabic, Greek, and Latin systems of traditional medicine [2]. These official monographs cite the characteristics and uses of the different parts of the plant, mainly the seeds. The fenugreek seed is widely known for its diverse phytochemical profile due to the presence of metabolites that impart unique medicinal, food, and nutritional properties to the seeds [3]. The phytochemical classes such as phenolics and flavonoids are widely known for their protective and therapeutic role against various types of human cancers due to the presence of functional groups with an ability to bind the signaling proteins in order to inhibit their prospective actions. The phytochemicals recognized for the aforementioned activities consist of saponins (diosgenin), flavonoids (quercetin, kaempferol, luteolin, and rutin), alkaloids (trigonelline), coumarins, vitamins, soluble fibers, and carbohydrates like galactomannan [4]. A plethora of literature reviews are available for fenugreek exploring the widely covering range, highlighting the huge potential the plant possesses for the development of new drugs. The prevalent scope may include its antioxidant, hypocholesterolemic, antineoplastic, anti-inflammatory, hepatoprotective, antiulcerogenic, antipyretic, immunomodulatory, and antitumor properties. Fenugreek seeds may also have a potential role in diabetes management, as they have been found to contain polyphenolic flavonoids, evident for its hypoglycemic, hypocholesterolemic, hypotriglyceridemics, and antiperoxidative effects [4]. Likewise, the presence of galactomannan, trigonelline, and diosgenin have been noticed with significant effects on diabetes through a number of different physiological pathways, e.g., the reduction in carbohydrate absorption, inhibition of alpha-amylase and sucrase activities, and restoration of pancreatic β-cell function [2]. A recent study revealed a lower fasting and post-prandial blood glucose levels for diabetic patients using the powder from fenugreek supplement for three months [5]. Fenugreek seeds have been reported to arrest the cancer cell cycle, inhibit cell proliferation in tumors, and reduce cell migration rates [2]. The selective cytotoxic effects of fenugreek seeds have been documented in various cancer cell lines including the colonic, pancreatic, prostate, breast, and T-cell lymphoma [1]. In addition, all parts of the fenugreek plant have demonstrated antimicrobial properties against bacteria such as *Pseudomonas aeruginosa*, *Escherichia coli*, *Staphylococcus aureus*, *Helicobacter pylori*, and *Rhizoctonia solani* [4]. Studies suggest the antibacterial role of fenugreek seed extracts is principally due to the naturally occurring combination of alkaloids, flavonoids, tannins, saponins, terpenoids, and steroids [5].

It is noteworthy to mention that a plethora of the literature has been reported with regard to the characterization and extraction of different phytochemical classes present in fenugreek seeds such as flavonoids, phenolics, alkaloids, saponins, and flavonoids glycosides [6,7,8,9,10,11]. In addition, a number of studies have reported the various biological and pharmacological potentials of fenugreek seeds in the prevention and treatment of inflammation, diabetes, cardiovascular diseases, hypercholesteremia, bacterial and fungal infections, cancer, and to boost a mother’s milk [12,13,14,15,16]. However, a research gap in the shape of a comprehensive correlation for the biological and pharmacological potential vs. the phytochemical classes of fenugreek seeds responsible for the reported activities still exists. There is an intense need to correlate the different phytochemical classes of the fenugreek seeds with the respective biological activities. This may further explore the phytochemical class and chemical components thereof, playing a vital role in the health-related activities of the fenugreek seeds.

The concept presented in the current study deal with the phytochemical analysis of the fenugreek seeds vs. the correlation with the biological activities of cytotoxicity, antibacterial, antifungal, and α-amylase conducted in-house. The detailed studies for the phytochemical classes of flavonoids and alkaloid were performed previously as reported [6,17], followed by the biological activities as mentioned. The statistical models were implicated to extract and highlight the significant correlations among the phytochemical classes and biological activities performed for fenugreek. The study recruited forty-five different origins of fenugreek seed samples in order to broaden the data pool for an effective statistical application.

There is a significant correlation between the phytochemical composition (saponins, phenolics, flavonoids, and alkaloids) of fenugreek seeds and their biological activities, including cytotoxicity, antibacterial, antifungal, and α-amylase inhibition. This study aims to investigate the phytochemical composition of fenugreek seeds from diverse origins and analyze their correlation with biological activities (cytotoxicity, antibacterial, antifungal, and α-amylase inhibition) using statistical models. The findings will contribute to understanding the functional potential of fenugreek phytochemicals in relation to their bioactivities.

## 2. Materials and Methods

### 2.1. Strains, Cells, Cell Culture Media, and Standard Drugs/Chemicals Used

The strains used in this study consisted of *Streptococcus faecalis* (ATCC*29212), *Staphylococcus aureus* (ATCC*29213), Acinetobacter baumannii (ATCC*1605), *Pseudomonas aeruginosa* (ATCC* 15442), and *Candida albicancs* (ATCC* 14053). Muller Hinton agar (Oxoid, Hampshire, United Kingdom, CM0337), nutrient agar, blood agar, Sabouraud dextrose agar, Sabouraud dextrose broth, and Muller Hinton broth (Oxoid, CM0405) were used for culture and susceptibility testing (diffusion method and broth dilution method) to determine the minimum inhibitory concentration (MIC) and minimum bactericidal concentrations (MBCs). The human breast adenocarcinoma (MCF-7; ATCC-HTB22), human colon adenocarcinoma (HT-29; ATCC- HTB-38™), hepatocellular carcinoma (HepG2; ATCC HB-8065), and normal human fetal lung fibroblast (MRC5; ATCC-CCL171) were tested in this study. The cell lines of MCF7, HT-29, and MRC5 were maintained in Roswell Park Memorial Institute Medium (RPMI-1640), whereas the HepG2 cell line was cultured in Dulbecco’s Modified Eagle Medium (DMEM) obtained from Gibco, Life Technologies, Carlsbad, CA, USA. The heat-inactivated fetal bovine serum (FBS) and antibiotics (1% penicillin-streptomycin) were also purchased from Gibco, Life Technologies, Carlsbad, CA, USA. MTT (3-(4,5-dimethylthiazol-2-yl)-2,5-diphenyltetrazolium bromide; Invitrogen, MA, USA) alpha-amylase (Sigma-Aldrich, MA, USA), Acarbose (Sigma-Aldrich), Paclitaxel (MedChemExpress, NJ, USA), Oxaliplatin (MedChemExpress), and Olaparib (MedChemExpress) were used.

### 2.2. Collection, Extraction, and Analysis for Fenugreek Samples

The fenugreek seed samples from five different origins were collected and extracted in three different solvents. The extracts were dried and the yield were calculated as reported in the previous studies [6]. The samples were subjected to flavonoids [6] and trigonelline [17] quantification using the in-house developed UHPLC-DAD analytical methods.

### 2.3. Cell Culture Studies

For cell culture studies, the three cell lines of MCF7, HT29, and MRC5 were maintained in the RPMI1640 medium whereas the HepG2 cell line was cultured in DMEM. The media were supplemented with 10%FBS and 1% penicillin-streptomycin antibiotic (10,000 units of penicillin +10,000 µg of streptomycin) and maintained at 37 °C in 5% CO_2_ with 100% relative humidity.

#### Determination of Cytotoxicity and Selectivity

Previously reported MTT assay was used to determine the cytotoxicity of the extracts in MCF7, HT29, HepG2, and MRC5 cell lines [18]. Briefly, the cells were cultured in 96-well plates and incubated (37 °C) overnight in order to screen the cytotoxic potential for the extracts at a concentration of 100 μg/mL (DMSO 0.4%; *n* = 3). The plates were kept for 48 h (37 °C in 5% CO_2_) followed by the addition of MTT and further incubation for 3 h. The supernatant was discarded whereas the MTT crystals were solubilized via the addition of DMSO to each well. The absorbance was recorded using a multi-plate reader (BIORAD, PR 4100, Hercules, CA, USA). The inhibition percentage compared to the control cells was calculated and the extracts with more %inhibition were selected for cytotoxicity determination (IC_50_ determination) in fibroblasts cell lines. The extract concentration studied ranged from 1 to 500 μg/mL (500, 250, 100, 50, 10, 1) whereas Paclitaxel, Oxaliplatin, and Olaparib were used as positive controls in order to determine the IC_50_ values using GraphPad Prism (San Diego, CA, USA).

### 2.4. Antimicrobial Studies

#### 2.4.1. Preparation of Standard Inoculum

The tested bacterial strains were grown on Muller Hinton (MH) agar medium whereas *Candida albicans* was grown on Sabouraud dextrose (SD) agar (37 °C; 24 h). To prepare inoculum, the selected colonies were inoculated in the MH and SB broth to form a homogenous suspension for the tested strains, standardized to 0.5 McFarland turbidity using a calibrated Vitek Densichek Biomerieux Analyzer (bioMérieux, Marcy-l’Étoile, France).

#### 2.4.2. Diffusion Assay on Agar Plates

For the agar well diffusion assay, tested strains (100 μL) with 0.5 McFarland turbidity were swabbed on the agar plate surfaces as per the reported protocol [19]. The plates were dried (10 min), and the wells were penetrated (6 mm) and filled with 100 µL of the tested extract (50 mg/mL) as well as positive (Vancomycin and Teicoplanin; 30 µg disks) and negative controls (Amikacin 30 µg and Imipenem 10 µg disks) for the bacterial strains. The solvent of dimethyl sulfoxide (DMSO) was used as a negative control. The process was performed in triplicate and the agar plates were incubated (24 h; 37 °C), examined, and zones of inhibition (mm) were measured.

#### 2.4.3. MIC and MBC Determination

The extracts with a significant zone of inhibitions were further assessed for MIC and MBC where a 96-well microtiter plate was used to prepare two-fold dilutions for the extracts at 50, 25, 12.5, 6.2, and 3.1 mg/mL. The 0.5 McFarland turbidity standards (10 μL) for *S. faecalis*, *S. aureus*, *A. baumannii*, *P. aeruginosa*, and *C. albicans* were poured into the wells of the selected samples and positive controls. The plates were incubated overnight (37 °C) followed by calculation of the MIC and MBC as per the guidelines of the clinical and laboratory standards institute [20,21].

### 2.5. Alpha-Amylase Inhibition Activity

A previously reported method was adopted for α-amylase inhibition [22]. Initially, the extracts were screened at a concentration of 500 μg/mL where the samples with significant α-amylase enzyme inhibition activity were further investigated in the concentration range of 1000, 500, 250, 100, 50, 25, and 5 μg/mL. The positive standard used was Acarbose. An aliquot of *Aspergillus. oryzae*-based α-amylase (1 mg) in phosphate buffer was prepared. To each well, 20 μL was added along with the extract samples (20 μL), mixed properly, and incubated for 10 min (37 °C). Following the incubation, 30 μL of the starch (0.05% in deionized water) solution was added to individual wells and incubated further for 8 min at 37 °C. The reaction was halted by the addition of 20 μL of hydrochloric acid (1 M) followed by 100 μL of the iodine reagent (0.25 mM) into each well. The control wells were prepared by replacing the enzyme with buffer solution and addition of Acarbose. The data were recorded by measuring the absorbance at 550 nm using a multi-plate reader (BIORAD, PR 4100, Hercules, CA, USA). The %inhibition was calculated for the wells using the formula

% inhibition = (*A − C/B** − C***) × 100

* absorbance of the reaction mixture in the presence of the extract, ** absorbance of the mixture without the enzyme, and *** absorbance of the reaction mixture in the absence of any extract.

### 2.6. Statistical Analysis

The results for the dataset were entered into an SPSS (statistical package for social science students V27.0) software and statistical models for the correlation and paired differences were applied. The descriptive data, i.e., mean ± standard deviation (SD), were generated from triplicate readings. The GraphPad Prism 9.2.0 (GraphPad, San Diego, CA, USA) software was used to determine the significant differences for the control vs. standard groups at *p* < 0.05.

## 3. Results

### 3.1. Cytotoxicity Results

The extracts with more %inhibition for HT29 constructed a descending order of cell viability: 11 ± 0.08 (IR3M) > 17 ± 0.06 (Q1M) > 19 ± 0.13 (IR2M) > 21 ± 0.10 (E2M) > 28 ± 0.14 (E1M) > 33 ± 0.11 (Y1M) > 42 ± 0.05 (E3M). For MCF7, the descending order observed for cell viability (%) was 9 ± 0.05 (IR2M) > 12 ± 0.05 (IR3M) > 13 ± 0.08 (Y1M) > 24 ± 0.19 (E2M) > 30 ± 0.11 (E1M) > 37 ± 0.09 (Q1M) > 44 ± 0.05 (E3M). The remaining samples for the fenugreek extracts showed more cell viability, i.e., the cell inhibition for the HT29 and MCF7 cell lines was >50%. The data for cell viability (%) are presented in Table 1.

#### Determination of IC_50_ Values for Cytotoxicity

The IC_50_ values calculated for the effective extracts of E1M, E2M, E3M [Egypt origin samples], Q1M [Saudi origin sample], Y1C [Yemen origin sample], IR2M, and IR3M [Iran origin samples] exhibited the lowest IC_50_ values (µg/mL) of 14.7 ± 1.46 for IR3M [Iran origin sample] against the HT29 cell line, 13.03 ± 1.95 for IR3M against the MCF7 cell line, 14.58 ± 2.92 for IR3M against the MRC5 line, and 8.17 ± 0.73 for IR2M against HepG2. The standard drugs used for comparative IC_50_ value calculations were oxaliplatin, olaparib, and paclitaxel. The data for the IC_50_ values against HT29, MCF7, MRC5, and HepG2 are shown in Table 2.

### 3.2. Antimicrobial Activity Results

The antimicrobial activity for fenugreek extracts against SF revealed a significant zone of inhibitions (ZOI: mm) for E2M (16) > E3C (15) > E2C (14). The samples of E1H, E1C, E1M, E2H, E3M, I1H, I3H, Q2H, and IR2H were seen with ZOI in the range of 10–13 mm, whereas the remaining samples were inactive to inhibit the growth of SF. For SA, the fenugreek extracts with significant results were E1M (25 mm) > E2C (24 mm) > E2H (22 mm). The extracts of I2C, I3H, Q2H, Y1C, Y3H, Y3C, Y3M, IR1H, IR2H, IR3H, and IR3C exhibited the ZOI in the range of 11–15 mm, whereas the remaining samples showed a lack of activity against SA. For antimicrobial activity against AB, only the Y2M sample (13 mm) was seen, whereas the inhibition against PA was seen for Q3H (12 mm) and Y2M (12 mm) only. None of the samples showed any inhibition for CA. The ZOIs for the fenugreek extracts against SA, SA, AB, PA, and CA are given in Table 1.

#### Determination of MIC and MBC

The MIC showed the lower value of 6.3 (mg/mL) for E2C, E2M, E3C [Egypt origin samples] and I3H [Indian origin sample] extracts against SF and for the E1M [Egypt origin sample], Y3C [Yemen origin sample], IR2H, IR3H, and IR3C [Iran origin samples] extracts against SA.

The MBC values exhibited the lower value of 12.5 (mg/mL) for E2C, E2M, E3C [Egypt origin samples] and I3H [Indian origin sample] against SF compared to samples E1M [Egypt origin sample], Y3C [Yemen origin sample], IR2H, IR3H, and IR3C [Iran origin samples] against SA. The data for the MIC and MBC are shown in Table 3.

### 3.3. Alpha-Amylase Inhibition Results

The results for α-amylase inhibitory activity (%) for the fenugreek extracts showed a descending order: 89 ± 0.10 (Y3C) > 89 ± 0.16 (Q1H) > 83 ± 0.03 (Y2C) > 77 ± 0.08 (I3C) > 77 ± 0.21 (Q3C) > 77 ± 0.13 (E3H) > 64 ± 0.22 (I2C) > 63 ± 0.17 (I1C) > 61 ± 0.24 (E1C) > 54 ± 0.07 (E3C). The remaining samples were seen with α-amylase inhibition of <50%. The data for α-amylase %inhibition for all the fenugreek extracts are shown in Table 1.

#### Determination of IC_50_ Values (µg/mL) for α-Amylase Inhibition

The IC_50_ values (µg/mL) for the extracts with inhibition >50% showed a comparatively significant activity for E3H (51.90 ± 2.29), I3C (52.40 ± 2.03) and Y2C (53.99 ± 2.49) with the standard drugs berberine and chlorogenic acid. The Q1H (49.07 ± 2.45) and Y3C (43.65 ± 2.97) extracts exhibited more potential inhibition for alpha-amylase. The IC_50_ values for the tested extracts are shown in Table 4.

### 3.4. Statistical Models

#### 3.4.1. Descriptive Statistics

For the biological activities, the cytotoxicity for HT29 and MCF7 (*n* = 45) exhibited the mean ± SD of 43.38 ± 28.86% and 33.73 ± 24.86% in the range (minimum–maximum) of 0–89%, and 2–91%, respectively. The alpha-amylase activity was seen with a mean (%) ± SD of 40.70 ± 19.72 in the range (minimum–maximum) of 17–89.

The antimicrobial results showed the mean (mm) ± SD (*n* = 45) with a range (minimum–maximum) of 3.49 ± 5.89 and 0–16 for SF, 4.67 ± 7.47 and 0–25 for SA, 0.29 ± 1.94 and 0–13 for AB, and 0.53 ± 2.50 and 0–12 for PA. The CA showed a lack of antimicrobial activity.

The descriptive results for the flavonoids were seen with a mean (ppm) ± SD and range (minimum–maximum) of 66.87 ± 143.02 and 0–499.1 (RT), 10.33 ± 34.02 and 0–212.0 (QT), 1.91 ± 3.32 and 0–14.1 (LT), and 0.36 ± 1.88 and 0–11.9 (KF).

For the alkaloid TG, a mean (ppb) ± SD of 181.37 ± 176.37 was observed in the range (minimum–maximum) of 22–535. The data for the descriptive statistics are provided in Table 5.

#### 3.4.2. Correlation Studies

The Pearson’s model was used to highlight the significant correlations for the biological activities of the fenugreek seed extracts vs. the phytochemical classes of flavonoids (FV) and alkaloid (AL). A high positive correlation was noted for the cell line activity (HT29 and MCF7: 0.66, *p* = 0.00) which was correlated with TG (0.52, *p* = 0.00). For the antimicrobial activity, SA and SF exhibited a positive correlation (0.322, *p* = 0.03). the species of AB and PA represented a positive pair with a correlation of 0.70, *p* = 0.00. All the flavonoids showed a high positive correlation with TG for the biological activities. The Pearson’s correlation data for the biological activities vs. phytochemical classes of the fenugreek seeds extracts are shown in Table 6.

#### 3.4.3. Principle Component Analysis (PCA)

**Figure 1:**
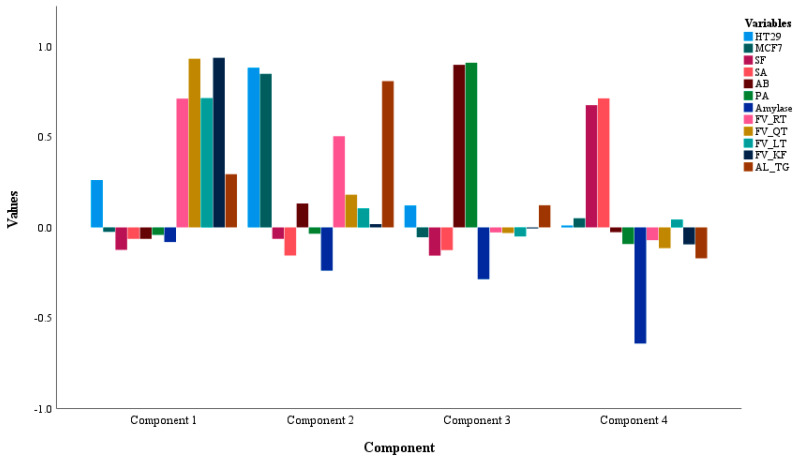
The loading of the DVs in different components for the fenugreek extract samples.

The principal component model was executed to analyze the variability of the dependent variables (DVs) within the groups. The scree plot suggested four components with a cumulative variance of 72.83% and individual variances of 24.58% (PC1), 21.26% (PC2), 14.94% (PC3), and 12.05% (PC4). The DVs loaded in PC1 were FV_ (RT, QT, LT, and KF), which suggest more %variance, i.e., a high positive inter-correlation for these activities vs. phytochemical classes. Following PC1, the next component with more %variability was PC2 (21.26) where the DV loaded was AL_TG with HT29 and MCF7. PC3 (14.94%) was loaded with AB and PA whereas, SF, SA, and amylase were loaded in PC4. The model was seen as significant at *p* = <0.001, a high *X*^2^-value of 298.141, and KMO-Bartlett’s test value of 0.602. The details regarding the PCA model are given in Table 7 and the loading for the DVs in the respective components is shown in Figure 1.

#### 3.4.4. K-Mean Analysis

The K-mean statistical model is applied to distribute a dataset into various clusters based on the similarities among the DVs. Herein, ten different clusters were suggested with the number of samples in each cluster as cluster 1 (7), cluster 2 (15), cluster 3 (4), cluster 4 (6), cluster 5 (8), cluster 6 (1), cluster 7 (1), cluster 8 (1), cluster 9 (1), and cluster 10 (1).

**Figure 2:**
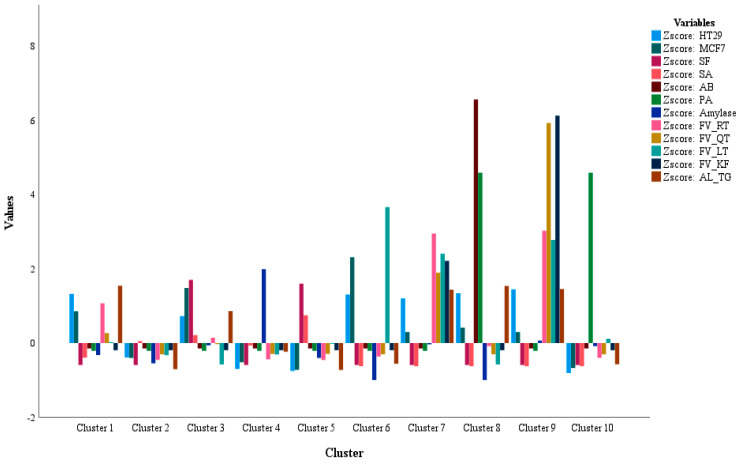
Distribution of the DV for fenugreek seed extracts in various clusters.

More HT29 and MCF7 activity was observed in sample loaded in cluster 6: IR2M [Iranian origin extracted in MeOH]. This sample was seen with the highest amount of LT. cluster 7 consisted of the sample I2M [Indian origin extracted in MeOH] with moderate HT29 and MCF7 activity where the presence for all the studied phytochemicals of FV and AL was noted in the sample. The highest antimicrobial activity for AB and PA alongwith a moderate activity for HT29 and MCF7 was observed for sample Y2M [Yemen origin extracted in MeOH] as shown in cluster 8. This sample was noted with the highest amount of TG. Cluster 9 was loaded with sample I3M [Indian origin extracted in MeOH] which is enriched with the highest amount of RT, QT, LT and KF alongwith a moderate amount of TG. This sample was seen with a good HT29 and MCF7 activity as well as a minor activity for amylase inhibition. The sample Q3H [Saudi Origin extracted in n-hexane] with the highest antimicrobial activity against PA was seen in cluster 10 where a small amount of LT was observed in the sample. The samples of E3H [Egyptian origin extracted in n-hexane], I2C & I3C [Indian origin extracted in chloroform], Y2C & Y3C [Yemen origin extracted in chloroform], and Q1H [Saudi origin extracted in n-hexane] with the highest inhibition for amylase activity were loaded in cluster 4. The fenugreek extracts in the remaining clusters presented a scattered pattern for the activities along with a low correlation with the phytochemicals. The F- and *p*-values for each DV observed in the K-mean model are given in Table 7, whereas the distribution of the DVs in various clusters is shown in Figure 2.

#### 3.4.5. Paired Samples *t*-Test

The paired samples *t*-test module was used to compute the differences between different pairs of the flavonoids and alkaloids vs. the biological activities of cytotoxicity, antimicrobial, and α-amylase inhibitory activity. The pairs for HT29, MCF7, and α-amylase inhibitory activity vs. the phytochemicals classes revealed no significant difference for the FV_RT (*p* > 0.05). For PA, no significant differences were found for FV_QT and FV_KF (*p* > 0.05). The activity against SF revealed no significant differences for FV_QT and FV_LT (*p* > 0.05), whereas for AB the only pair with a lack of significant difference in activity was FV_KF. All the remaining pairs for HT29, MCF7, amylase, PA, SF, SA, and AB exhibited significant differences with regard to the biological activities suggesting a positive role for the flavonoids and alkaloids in the biological activities of fenugreek seeds. The data for the paired differences in the biological activities vs. phytochemical classes of the fenugreek seed extracts are given in detail in Table 8.

## 4. Discussion

Fenugreek seeds are acknowledged for their diverse biological activities associated with the presence of a number of phytochemical classes. This research study aims to establish a potential correlation for the key phytochemical classes of flavonoids and alkaloids reported in the fenugreek seeds with the cytotoxicity, antimicrobial, and α-amylase inhibitory activity tested herein. The extracts for the fenugreek seeds put into use for the biological activities were extracted in our previous study [6]. To assess the cytotoxicity potential for the fenugreek seed extracts, HT29, MCF7, MRC5, and HepG2 cell lines were utilized where the cell lines of HT29 and MCF7 were tested for the preliminary cytotoxicity potential of the extracts. The idea was to extricate the fenugreek samples with a potential to inhibit the cell viability with an initial checkup value of >50%. The extracts of E1M, E2M, E3M, Y1M, IR2M, IR3M, and Q1M were seen with an inhibition of 50% or above for both the cell lines of HT29 and MCF7. Hence, these extracts were selected for further testing in order to determine the IC_50_ values for these sample. To investigate the selectivity and observe the cell lines coverage for these extracts, HepG2 and MRC5 cell lines along with six further MTT concentrations were included for IC_50_ determination. The extracts of IR2M (HepG2) and IR3M (HT29, MCF7, and MRC5) showed significantly lower IC_50_ values compared to the other extracts. The samples with cytotoxic potential observed herein were the methanol extracted seeds of fenugreek. Previous studies revealed a dose-dependent cytotoxic effect and apoptosis induction for the methanolic extract of the fenugreek seeds against MCF-7 breast and HepG2 hepatocellular carcinoma cell lines [23,24]. These results corroborate the findings for cytotoxicity observed in our study.

The antimicrobial potential for the fenugreek seed extracts was studied against the bacterial (SF, SA, AB, and PA) and fungal strains (CA). The extracts exhibited potential antibacterial activity against SF (E2M, E3C, and E2C), SA (E1M, E2C, and E2H), AB (Y2M), and PA (Q3H and Y2M); however, a lack of antifungal activity was recorded for all the extracts against CA. The results from the previous studies presented a potential effect for fenugreek extract against Gram-positive (*S. aureus*) and Gram-negative bacteria (*P. aeruginosa*) [25]. The literature herein supports the prospective role for the antibacterial uses of fenugreek seeds. The selected samples were tested for MIC and MBD determination. The data showed the lowest MIC against SF (E2C, E2M, E3C, I3H), SA (E1M, Y3C, IR2H, IR3H, IR3C) as well as the MBC against SF (E2C, E2M, E3C, I3H) and SA (E1M, Y3C, IR2H, IR3H, IR3C).

The α-amylase inhibition for the extracts was studied where an initial screening at a dose of 500 μg/mL for each fenugreek extract was employed to acquire the extracts with an α-amylase inhibitory potential of >50%. The result yielded the Y3C, Q1H, Y2C, I3C, Q3C, E3H, I2C, I1C, E1C, and E3C extracts with an inhibition of >50% for α-amylase. The mentioned extracts were selected and studied further at six different low-dose concentrations to calculate the IC_50_ values. The extracts of E3H, I3C, Q1H, YC, and Y3C resulted in the lower IC_50_ values comparable to the standard drugs tested in the study. The ability of the fenugreek seeds to modify and inhibit the carbohydrate-metabolizing enzymes (α-amylase) in a dose-dependent manner have been reported previously [26,27]. The reported studies demarcate the antidiabetic role for fenugreek seeds as observed in current study.

The statistical models were applied to link the significant correlations and paired differences among the biological activities vs. the phytochemicals analyzed for these extracts. The Pearson’s analysis revealed a high positive correlation for the inhibitory effect upon the HT29 and MCF7 cell lines with the extracts possessing the phytochemical classes of flavonoids (RT, QT, LT, and KF) and the alkaloid TG. As well as cytotoxicity, the α-amylase inhibitory activity was noticed in the extracts containing a significant amount of the flavonoids and alkaloid. Likewise, the antimicrobial activity against SF and SA was recognized in the fenugreek extracts containing more amounts of TG and LT. To comprehend the correlation link between the phytochemical classes and biological activities, component analysis was performed to bring out the variables with less inter-variability, i.e., more correlation, loaded in the same components. As evident from the PCA data, the cytotoxicity and α-amylase activities were loaded alongside the flavonoids and alkaloid whereas the variables for antimicrobial activity were loaded in one cluster, suggesting a prominent role for the analyzed phytochemical classes in the biological activities of fenugreek seeds. However, the sample pool was larger with 45 different fenugreek seed extracts, hence K-mean analysis was utilized to highlight the seed extracts with more phytochemical amounts and higher activity. Interestingly, K-mean analysis clustered the extracts with the most potent biological activities containing a significant amount of either flavonoids and alkaloid, or altogether. For instance, the designated extracts (phytochemical class) were: IR2M [Iranian origin] with more cytotoxicity against MCF7 and HT29; I2M [Indian origin] with moderate HT29 and MCF7; Y2M [Yemen origin] with the highest antimicrobial activity against AB and PA alongwith a moderate activity for HT29 and MCF7; I3M [Indian origin] with a significant activity against HT29 and MCF7 activity as well as a minor activity for amylase inhibition; Q3H [Saudi Origin] with the highest antimicrobial activity against PA; E3H [Egyptian origin], I2C & I3C [Indian origin], Y2C & Y3C [Yemen origin], and Q1H [Saudi origin] with the highest inhibition for amylase activity.

The K-mean cluster activity was further confirmed by the paired sample *t*-test. The paired differences for the phytochemical classes of flavonoids (QT, LT, KF, and AG) and alkaloid (TG) are shown in the table where the differences observed confirms an alike pattern for the correlation pattern seen in the statistical models of Pearson’s correlation, PCA, and K-mean cluster analysis.

The variation in the phytochemical composition of the fenugreek seeds and its biological activities thereof are prone to a number of factors including the geographical variation where the factors of humidity, salinity, altitude, soil composition, temperature, water irrigation, soil water retention capacity, annual rainfall level, and pH of the soil play a substantial role. In addition, the harvesting time, seeds processing, shipment, and its storage are the key factors to be considered for preserving the optimal quality of the seeds or plants [28,29].

These phytochemical classes owe the potential to inhibit the initiation of carcinogenesis via modulation of the phase I and II carcinogen-metabolizing enzymes as well as inhibition of oxygen radical-forming enzymes that contribute to DNA synthesis and inhibition of protein kinases playing a role in the proliferative signal transduction and cell cycle. The flavonoids suppress tumor development through the induction of cellular apoptosis mediated via suppression of DNA topoisomerase and p53, induction of autophagy, inhibition of telomerase, and triggering of mitochondrial toxicity [30,31].

Likewise, the phytochemical class of alkaloids is well known for its antioxidant, anticancer, and antidiabetic medicinal uses. Alkaloids are accepted as one of the richest and most productive sources of drug discovery, particularly with regard to cancer treatments. The in vitro and in vivo experiments have revealed the potential anticancer properties of alkaloids, mediated through interactions with various aspects of tumor progression. The alkaloids may induce caspase-dependent and caspase-independent apoptosis as well as modulate both the pro-apoptotic BAX/BAK and anti-apoptotic BCL-2 protein expression. They also regulate autophagy through the modulation of phosphatidylinositol 3-kinase, mitogen-activated protein kinases, and reactive oxygen species. In addition, they arrest the cell cycle at different stages through the regulation of a cyclin-dependent kinase family of proteins. They suppress angiogenesis, invasion and metastasis in a variety of cancer cell lines through inhibition of focal adhesion kinase, vascular endothelial growth factor, and matrix metalloproteinase [32,33]. The research work herein, crosslinking the statistical data for the biological activities vs. the phytochemical classes of flavonoids and alkaloid analyzed in our studies, elucidate a substantial role for these phytochemical classes in the health care applications of fenugreek seeds in terms of antioxidant, anticancer, antimicrobial, and as an antidiabetic. The support from the previous literature data favor the presence of flavonoids and alkaloids being the key components behind the traditional as well as the biological role of fenugreek seeds.

## 5. Conclusions

This research work provides a comprehensive correlation for the multiple classes of fenugreek seed extracts (flavonoids and alkaloid) in establishing its role in cytotoxicity, antimicrobial, and antidiabetic activity (α-amylase inhibition). The in-depth statistical analysis for the multiple classes of the fenugreek seed extracts substantiates a significant role for the flavonoids (QT, LT, KF, AG) and alkaloid (TG) to inhibit the cell growth for MCF7 and HT29, microorganisms (SF, and SA), and α-amylase activity. Herein, the study establishes the pharmacological role for the fenugreek seeds to be properly correlated with the traditional uses of fenugreek. The samples of IR2M, IR2 and 3H, IR2 and 3H, IR3C [Iran origin samples], E2 and 3C, E1 and 2M [Egypt origin samples], I3H [Indian origin], and Q1H [Saudi origin] were more effective for the biological activities. This study may be helpful for the quality control and standardization of fenugreek to evaluate and authenticate the fenugreek seeds as well as the pharmaceutical products and dietary supplements for the presence of the essential key elements of flavonoids and alkaloid. The major insight that the current study provides is the identification and correlation of the fenugreek phytochemical class and its respective chemical moiety responsible for the claimed medicinal uses; hence, this paves a way for researchers to isolate and utilize the same chemical moiety for the established medicinal use. The identified compounds may be utilized as biomarkers for plant-based drug discovery and bioengineering to open avenues for genetic modification in order to enhance the yield for the phytochemical compounds. The future directives may include in-depth study for the geographical variation affecting the metabolites’ number and concentration. In addition, the identified metabolites may be enhanced for their yield within the same source, utilizing the genetic biodiversity and gene manipulations technique.

## Figures and Tables

**Table 1:** Cytotoxicity, antimicrobial, and α-amylase results for fenugreek seed extracts. H (hexane extract), C (chloroform extract), M (methanol extract). Origins (E, Egypt; I, India; Q, Saudi Arabia; IR, Iran; Y, Yemen). ^&^ R (resistant). * SF (*Streptococcus faecalis*; ATCC*29212), SA (*Staphylococcus aureus*; ATCC*29213), AB (*Acinetobacter baumannii*; ATCC*1605), PA (*Pseudomonas aeruginosa*; ATCC*15442), CA (*Candida albican*; ATCC*14053).

S#	Origin	Collection Area/City	Sample ID	Cytotoxicity (%)		Antimicrobial (mm)					α-Amylase Inhibition (%)	Flavonoids (ppm)				Alkaloid (ppb)
				HT29	MCF7	SF	SA	AB	PA	CA		RT	QT	LT	KF	TG
1	Egypt	Jeddah	E1H	99 ± 0.06	82 ± 0.12	13	^&^ R	R	R	R	37 ± 0.04	-	-	0.59	-	100.7
2			E1C	52 ± 0.18	20 ± 0.15	13	R	R	R	R	61 ± 0.24	4.66	-	-	-	107.5
3			E1M	28 ± 0.14	30 ± 0.11	13	25	R	R	R	38 ± 0.12	109.40	-	-	-	391.0
4			E2H	89 ± 0.01	90 ± 0.17	12	22	R	R	R	33 ± 0.05	-	-	7.65	-	47.2
5			E2C	69 ± 0.04	92 ± 0.10	14	23	R	R	R	24 ± 0.10	-	-	-	-	35.4
6			E2M	21 ± 0.10	24 ± 0.19	16	R	R	R	R	27 ± 0.09	207.98	17.22	-	-	407.0
7		Riyadh	E3H	100 ± 0.16	80 ± 0.16	R	R	R	R	R	77 ± 0.13	-	-	2.10	-	91.6
8			E3C	71 ± 0.14	97 ± 0.09	15	R	R	R	R	54 ± 0.07	-	-	-	-	102.3
9			E3M	42 ± 0.05	44 ± 0.05	12	R	R	R	R	32 ± 0.12	28.00	14.73	-	-	423.0
10	India	Jeddah	I1H	86 ± 0.03	98 ± 0.04	13	R	R	R	R	25 ± 0.17	-	-	-	-	36.7
11			I1C	85 ± 0.05	95 ± 0.09	R	R	R	R	R	63 ± 0.17	-	-		-	108.1
12			I1M	12 ± 0.14	58 ± 0.12	R	R	R	R	R	46 ± 0.12	252.70	24.22	1.94	-	535.0
13		Riyadh	I2H	92 ± 0.18	91 ± 0.10	R	R	R	R	R	34 ± 0.11	-	-	-	-	56.6
14			I2C	71 ± 0.07	86 ± 0.09	R	13	R	R	R	64 ± 0.22	-	-	-	-	105.5
15			I2M	22 ± 0.14	59 ± 0.11	R	R	R	R	R	40 ± 0.13	488.28	74.66	9.90	4.54	434.0
16			I3H	79 ± 0.11	68 ± 0.07	13	14	R	R	R	34 ± 0.04	-	-	1.24	-	43.2
17			I3C	83 ± 0.16	90 ± 0.18	R	R	R	R	R	77 ± 0.08	-	-	-	-	146.0
18			I3M	15 ± 0.11	59 ± 0.09	R	R	R	R	R	42 ± 0.22	499.06	212.04	11.14	-	437.0
19	Saudi Arabia	Jeddah	Q1H	89 ± 0.10	81 ± 0.11	R	R	R	R	R	89 ± 0.16	-	-	-	-	34.3
20			Q1C	71 ± 0.09	82 ± 0.11	R	R	R	R	R	43 ± 0.17	-	-	1.28	-	50.1
21			Q1M	17 ± 0.06	37 ± 0.09	R	R	R	R	R	42 ± 0.20	-	10.11	-	-	510.0
22		Riyadh	Q2H	80 ± 0.05	92 ± 0.06	12	11	R	R	R	35 ± 0.18	-	2.61	5.41	-	37.0
23			Q2C	42 ± 0.17	79 ± 0.17	R	R	R	R	R	40 ± 0.24	-	-	-	-	46.7
24			Q2M	13 ± 0.05	53 ± 0.08	R	R	R	R	R	23 ± 0.22	445.51	53.87	5.30	-	491.0
25			Q3H	80 ± 0.16	83 ± 0.18	R	R	R	12	R	39 ± 0.18	-	-	2.30	-	80.4
26			Q3C	66 ± 0.15	46 ± 0.11	R	R	R	R	R	77 ± 0.21	0.04	-		-	62.3
27			Q3M	24 ± 0.19	85 ± 0.11	R	R	R	R	R	39 ± 0.12	208.87	27.20	-	-	405.0
28	Yemen	Jeddah	Y1H	77 ± 0.20	90 ± 0.09	R	R	R	R	R	38 ± 0.16	-	-	-	-	58.4
29			Y1C	19 ± 0.20	75 ± 0.12	R	12	R	R	R	17 ± 0.08	-	-	-	-	24.4
30			Y1M	33 ± 0.11	13 ± 0.08	R	R	R	R	R	28 ± 0.17	-	18.18	1.32	-	385.0
31		Riyadh	Y2H	100 ± 0.11	83 ± 0.16	R	R	R	R	R	30 ± 0.15	-	-	3.61	-	49.1
32			Y2C	54 ± 0.10	84 ± 0.17	R	R	R	R	R	83 ± 0.03	-	-	-	-	102.7
33			Y2M	18 ± 0.09	56 ± 0.05	R	R	13	12	R	21 ± 0.10	54.64	-	-	-	452.0
34			Y3H	86 ± 0.10	87 ± 0.10	R	12	R	R	R	32 ± 0.18	-	-	0.53	-	74.3
35			Y3C	64 ± 0.13	54 ± 0.07	R	12	R	R	R	89 ± 0.10	-	2.21	3.25	-	359.2
36			Y3M	20 ± 0.08	58 ± 0.02	R	12	R	R	R	30 ± 0.21	475.48	-	3.30	-	475.0
37	Iran	Jeddah	IR1H	53 ± 0.11	89 ± 0.15	R	13	R	R	R	26 ± 0.13	-	-	-	-	27.3
38			IR1C	54 ± 0.25	60 ± 0.14	R	R	R	R	R	20 ± 0.21	1.69	-	-	-	22.2
39			IR1M	82 ± 0.09	68 ± 0.22	R	R	R	R	R	24 ± 0.19	-	-	5.27	-	92.6
40			IR2H	54 ± 0.09	55 ± 0.35	11	12	R	R	R	20 ± 0.19	-	-	-	-	22.6
41			IR2C	73 ± 0.13	57 ± 0.08	R	R	R	R	R	24 ± 0.11	-	-	-	-	24.3
42			IR2M	19 ± 0.13	9 ± 0.05	R	R	R	R	R	21 ± 0.14	-	-	14.08	-	82.9
43		Riyadh	IR3H	75 ± 0.14	72 ± 0.11	R	15	R	R	R	32 ± 0.05	-	-	-	-	133.2
44			IR3C	58 ± 0.16	59 ± 0.13	R	14	R	R	R	29 ± 0.16	-	-	-	-	81.7
45			IR3M	11 ± 0.08	12 ± 0.05	R	R	R	R	R	32 ± 0.23	148.23	-	2.16	-	370.0

**Table 2:** IC50 calculation for the potent cytotoxic samples.

Sample	HT29	MCF7	MRC5	HepG2
E1M	27.56 ± 0.96	39.31 ± 2.13	48.84 ± 2.31	80.24 ± 0.65
E2M	26.96 ± 1.67	32.40 ± 2.68	31.23 ± 1.90	87.18 ± 2.39
E3M	54.93 ± 2.66	72.34 ± 0.84	66.63 ± 2.49	126.4 ± 1.41
Q1M	16.88 ± 1.63	48.64 ± 2.53	17.09 ± 3.05	14.57 ± 0.81
Y1M	34.68 ± 1.85	17.29 ± 0.54	82.20 ± 1.56	78.4 ± 2.70
IR2M	20.10 ± 2.63	15.06 ± 2.05	30.42 ± 3.56	8.17 ± 0.73
IR3M	14.7 ± 1.46	13.03 ± 1.95	14.58 ± 2.92	16.19 ± 2.79
Oxaliplatin	1.18 ± 0.25	4.29 ± 1.08	15.05 ± 1.88	4.57 ± 1.09
Olaparib	4.62 ± 0.49	2.44 ± 0.59	14.39 ± 1.37	8.62 ± 1.18
Paclitaxel	0.38 ± 0.06	0.40 ± 0.05	1.8 ± 0.03	6.9 ± 0.81

**Table 3:** MIC (mg/mL) and MBC (mg/mL) determination for the active fenugreek extracts.

S#	Code	*SF*		*SA*	
		ATCC*29212		ATCC*29213	
		MIC	MBC	MIC	MBC
1	Q3H	-	-	-	-
2	Y2M	-	-	-	-
3	E1H	12.5	25	-	-
4	E1C	12.5	25	-	-
5	E1M	12.5	25	6.3	12.5
6	E2H	12.5	25	12.5	25
7	E2C	6.3	12.5	12.5	25
8	E2M	6.3	12.5	-	-
9	E3C	6.3	12.5	-	-
10	E3M	12.5	25	-	-
11	I1H	12.5	25	-	-
12	I2C	-	-	12.5	25
13	I3H	6.3	12.5	12.5	25
14	Q2H	12.5	25	12.5	25
15	Y1C	-	-	12.5	25
16	Y3H	-	-	25	50
17	Y3C	-	-	6.3	12.5
18	Y3M	-	-	12.5	25
19	IR1H	-	-	12.5	25
20	IR2H	12.5	25	6.3	12.5
21	IR3H	-	-	6.3	12.5
22	IR3C	-	-	6.3	12.5

**Table 4:** IC50 values for active extracts (>50%) in α-amylase activity.

Code	IC_50_
E1C	139.7 ± 3.12
E3H	51.90 ± 2.29
E3C	201.2 ± 2.26
I1C	147.3 ± 2.03
I2C	123.0 ± 2.52
I3C	52.40 ± 2.03
Q1H	49.07 ± 2.45
Q3C	64.15 ± 3.26
Y2C	53.99 ± 2.49
Y3C	43.65 ± 2.97
Positive control (Berberine)	60.83 ±0.18
Positive control (Chlorogenic acid)	51.23 ± 0.22

**Table 5:** Descriptive statistics for the dataset. FV (flavonoids) and AL (alkaloid).

	Minimum	Maximum	Sum	Mean	SD
HT29	0	89	1952	43.38	28.86
MCF7	2	91	1518	33.73	24.86
SF	0	16	157	3.49	5.89
SA	0	25	210	4.67	7.46
AB	0	13	13	0.29	1.93
PA	0	12	24	0.53	2.50
CA	0	0	0	0.00	0.00
Amylase	17	89	1831	40.69	19.72
FV_RT	0	499.1	3009.3	66.87	143.03
FV_QT	0	212.0	465.0	10.33	34.02
FV_LT	0	14.1	82.4	1.91	3.32
FV_KF	0	11.9	16.5	0.36	1.88
AL-TG	22.2	535.0	8161.5	181.36	176.38

**Table 6:** Pearson’s correlation data for the biological activities vs. phytochemical classes of the fenugreek seed extracts.

	MCF7	SF	SA	AB	PA	Amylase	FV_RT	FV_QT	FV_LT	FV_KF	AL_TG
HT29	0.66	−0.14	−0.08	0.20	0.06	−0.29	0.62	0.41	0.22	0.27	0.75
	0.00	0.35	0.58	0.18	0.71	0.05	0.00	0.01	0.15	0.07	0.00
MCF7	1.00	0.01	−0.13	0.06	−0.03	−0.17	0.24	0.13	0.23	0.06	0.52
		0.93	0.41	0.68	0.85	0.26	0.11	0.38	0.15	0.71	0.00
SF	0.01	1.00	0.322 *	−0.09	−0.13	−0.17	−0.14	−0.12	−0.14	−0.12	−0.10
	0.93		0.03	0.56	0.40	0.27	0.35	0.43	0.36	0.44	0.50
SA	−0.13	0.322 *	1.00	−0.10	−0.14	−0.18	−0.11	−0.17	−0.07	−0.12	−0.16
	0.41	0.03		0.53	0.37	0.25	0.47	0.25	0.66	0.42	0.30
AB	0.06	−0.09	−0.10	1.00	0.70	−0.15	−0.01	−0.05	−0.09	−0.03	0.23
	0.68	0.56	0.53		0.00	0.32	0.93	0.76	0.57	0.85	0.12
PA	−0.03	−0.13	−0.14	0.70	1.00	−0.12	−0.05	−0.07	−0.05	−0.04	0.10
	0.85	0.40	0.37	0.00		0.44	0.73	0.67	0.74	0.78	0.49
Amylase	−0.17	−0.17	−0.18	−0.15	−0.12	1.00	−0.14	−0.04	−0.12	0.01	−0.05
	0.26	0.27	0.25	0.32	0.44		0.37	0.80	0.46	0.96	0.76
FV_RT	0.24	−0.14	−0.11	−0.01	−0.05	−0.14	1.00	0.71	0.48	0.59	0.70
	0.11	0.35	0.47	0.93	0.73	0.37		0.00	0.00	0.00	0.00
FV_QT	0.13	−0.12	−0.17	−0.05	−0.07	−0.04	0.71	1.00	0.53	0.95	0.46
	0.38	0.43	0.25	0.76	0.67	0.80	0.00		0.00	0.00	0.00
FV_LT	0.23	−0.14	−0.07	−0.09	−0.05	−0.12	0.48	0.53	1.00	0.54	0.18
	0.15	0.36	0.66	0.57	0.74	0.46	0.00	0.00		0.00	0.25
FV_KF	0.06	−0.12	−0.12	−0.03	−0.04	0.01	0.59	0.95	0.54	1.00	0.29
	0.71	0.44	0.42	0.85	0.78	0.96	0.00	0.00	0.00		0.06
AL_TG	0.52	−0.10	−0.16	0.23	0.10	−0.05	0.70	0.46	0.18	0.29	1.00
	0.00	0.50	0.30	0.12	0.49	0.76	0.00	0.00	0.25	0.06	

* indicates the correlation < 0.01.

**Table 7:** PCA and K-mean cluster analysis for the fenugreek seed extracts’ biological activities.

DV	1	2	3	4	KMO and Bartlett’s Test				
HT29	0.26	0.88	0.12	0.01	Kaiser–Meyer–Olkin Sampling Adequacy			0.602	
MCF7	−0.02	0.84	−0.05	0.05	Bartlett’s Test of Sphericity	Approx. Chi-Square		298.141	
SF	−0.12	−0.06	−0.15	0.67		df		66	
SA	−0.06	−0.15	−0.12	0.71		Sig.		<0.001	
AB	−0.06	0.13	0.89	−0.02					
PA	−0.04	−0.03	0.90	−0.09					
Amylase	−0.08	−0.23	−0.28	−0.64					
FV_RT	0.71	0.50	−0.02	−0.07					
FV_QT	0.93	0.18	−0.03	−0.11					
FV_LT	0.71	0.10	−0.05	0.04					
FV_KF	0.93	0.01	−0.006	−0.09					
AL_TG	0.29	0.80	0.12	−0.17					
Individual variance (%)	24.58	21.26	14.94	12.05					
Cumulative variance (%)	24.58	45.84	60.78	72.83					
Factors			F-value		Clusters		Samples		
Z-score: HT29			9.95		1		7		
Z-score: MCF7			7.20		2		15		
Z-score: SF			273.38		3		4		
Z-score: SA			0.79		4		6		
Z-score: AB			3.87		5		8		
Z-score: PA			6.48		6		1		
Z-score: amylase			14.80		7		1		
Z-score: FV_RT			9.76		8		1		
Z-score: FV_QT			79.94		9		1		
Z-score: FV_LT			9.72		10		1		
Z-score: FV_KF			57.98		Total		45		
Z-score: AL_TG			24.31						

**Table 8:** Paired sample test for the analysis of variance in the biological activities of the fenugreek extracts vs. phytochemical profiling.

Paired Sample Test					
Pairs	Variables	Mean	95% CI of the Difference		Sig
			Lower	Upper	
HT29 Paired Differences					
Pair 1	HT29-FV_RT	−23.49	−61.73	14.74	0.22
Pair 2	HT29-FV_QT	33.04	22.66	43.41	<0.001
Pair 3	HT29-FV_LT	42.34	33.53	51.14	<0.001
Pair 4	HT29-FV_KF	43.01	34.47	51.54	<0.001
Pair 9	HT29-AL_TG	−137.98	−184.85	−91.12	<0.001
MCF7 Paired Differences					
Pair 1	MCF7-FV_RT	−33.13	−74.92	8.64	0.18
Pair 2	MCF7-FV_QT	23.39	11.57	35.22	<0.001
Pair 3	MCF7-FV_LT	32.01	24.52	39.50	<0.001
Pair 4	MCF7-FV_KF	33.36	25.910	40.82	<0.001
Pair 9	MCF7-AL_TG	−147.63	−197.17	−98.11	<0.001
α-amylase Paired Differences					
Pair 1	α-amylase-FV_RT	−26.18	−70.35	17.98	0.24
Pair 2	α-amylase-FV_QT	30.35	18.33	42.37	<0.001
Pair 3	α-amylase-FV_LT	37.41	31.34	43.47	<0.001
Pair 4	α-amylase-FV_KF	40.32	34.37	46.27	<0.001
Pair 9	α-amylase-AL_TG	−140.67	−194.27	−87.08	<0.001
PA paired differences					
Pair 1	PA-FV_RT	−66.33	−109.35	−23.31	0.003
Pair 2	PA-FV_QT	−9.80	−20.10	0.50	0.062
Pair 3	PA-FV_LT	−1.35	−2.68	−0.003	0.045
Pair 4	PA-FV_KF	0.16	−0.079	1.12	0.727
Pair 9	PA-AL_TG	−180.83	−233.75	−127.91	<0.001
SA Paired Differences					
Pair 1	SA-FV_RT	−62.20	−105.48	−18.92	0.006
Pair 2	SA-FV_QT	−5.66	−16.50	5.17	0.298
Pair 3	SA-FV_LT	2.96	0.35	5.57	0.027
Pair 4	SA-FV_KF	4.30	1.91	6.68	0.001
Pair 9	SA-AL_TG	−176.70	−230.09	−123.39	<0.001
SF Paired Differences					
Pair 1	SF-FV_RT	−63.38	−106.64	−20.12	0.005
Pair 2	SF-FV_QT	−6.84	−17.42	3.73	0.199
Pair 3	SF-FV_LT	1.73	−0.49	3.96	0.124
Pair 4	SF-FV_KF	3.12	1.20	5.04	0.002
Pair 9	SF-AL_TG	−177.87	−231.08	−124.67	<0.001
AB Paired Differences					
Pair 1	AB-FV_RT	−66.58	−109.56	−23.59	0.003
Pair 2	AB-FV_QT	−10.04	−20.31	0.22	0.055
Pair 3	AB-FV_LT	−1.61	−2.85	−0.37	0.012
Pair 4	AB-FV_KF	−0.07	−0.90	0.74	0.851
Pair 9	AB-AL_TG	−181.07	−233.97	−128.21	<0.001

## Data Availability

The original contributions presented in this study are included in the article. Further inquiries can be directed to the corresponding author.
